# Supplementary material for: Toxoplasma gondii infection and high levels of IgE are associated to erythema nodosum leprosy (ENL)
Source: PLoS One. 2024 Jun 12;19(6):e0300704. doi: 10.1371/journal.pone.0300704 (PMC11168690; doi:10.1371/journal.pone.0300704)
Supplement: Attachment — Submitted filename: Response to Reviewers.pdf [file pone.0300704.s002.pdf]

GOVERNO DO ESTADO
RIO DE JANEIRO
Secretaria de Estado de Ciência, Tecnologia e Inovação
Universidade Estadual do Norte Fluminense Darcy Ribeiro
Centro de Biociências e Biotecnologia
Laboratório de Biologia do Reconhecer

Dr[a]. Alba Lucínia Peixoto Rangel
State University of Northern Rio de Janeiro
Bioscience and Biotechnology Center
Recognized Biology Laboratory
Alberto Lamego Avenue, 2000
Campos dos Goytacazes, RJ, Brazil
28013-602
alba@uenf.br

Drª Musa M. Ali
Academic Editor
Plos One

December 12, 2023

Dear *Plos One* Academic Editor:

I am pleased to submit our revised manuscript entitled "***Toxoplasma gondii* infection and high levels of IgE are associated to erythema nodosum leprosy (ENL)**" for publication as Research Article in the *Plos One*. Follow below our Response to Editor and Reviewers:

Editor Comments:

#We have received reviewers comments. Before reaching to the decision, we would like you to address all comments provided by the reviewers including why 95% CI was wide for ENL and T. gondii? Will it affect the interpretation of your finding?

The odds ratio should be expressed with a confidence interval calculated from a predetermined margin of error. The sample size does not affect the odds ratio but does affect its confidence interval. So, the larger the study sample, the smaller the confidence interval. If the aim is to measure a positive association between a potential risk factor and the disease event, as our study, the lower limit of the confidence interval is the crucial element. This limit should be higher than 1 for us to assert that there is an association within a given confidence interval. In our study, the data showing a confidence interval of 1.050 - 72.85 reflects a small sample size; however, it indicates an association between the risk factor and the disease, confirmed by a significant p-value and the lower limit of the confidence interval. So, answering your question, our wide 95% CI for ENL and *T. gondii* not affect the interpretation of our findings.

#And also respond to the issue of *M. leprae* infection in the foot of the BALB/c mouse mention in the discussion section.

Infection of the footpad of BALB/c nude mice with a viable *M. leprae* strain is a model used to maintain a viable *M. leprae* strain in laboratories. We ourselves make use of this *M. leprae* strain maintenance model in our laboratory. However, the authors of the cited work used an immunocompetent Balb/c mouse chronically infected with *T. gondii* to infect the footpad with a viable strain of *M. leprae* and observe the relationship of susceptibility and resistance related to the amount of *M. leprae* bacilli recovered from footpad. So, as this work referenced by us in the discussion was the only one in the scientific literature that showed a model of *M. leprae*/*T. gondii*, we use the authors' conclusion in our discussion. The phrase *"Contrary to our data, BALB/c mice chronically infected with the intracellular protozoan T. gondii or Besnoitia jellisoni were resistant to footpad challenge with M. leprae. Resistance was manifested by lower numbers of recoverable M. leprae in the footpads of protozoal-infected mice and resistance was enhanced in Toxoplasma-infected mice by a booster injection of Toxoplasma antigen in the infected footpad (Krahenbuhl JL, Levy L, Remington JS. Resistance to Mycobacterium leprae in Mice Infected with Toxoplasma gondii and Besnoitia jellisoni. Infect Immun. 1974 Nov;10(5):1068-71. doi: 10.1128/iai.10.5.1068-1071.1974. PMID: 16558091; PMCID: PMC423063.)"* used in our discussion was not our conclusion, it was from the authors of the work we cited. We transcribe their conclusion in the context of our discussion even using the same words.

#What kind of bacteriological examination was made for classification assuming it is difficult to grow *M. leprae* in artificial culture media.

Following WHO determination (World Health Organization. Regional Office for South-East Asia. (2018). Guidelines for the diagnosis, treatment and prevention of leprosy. World Health Organization. Regional Office for South-East Asia. https://iris.who.int/handle/10665/274127. Licença: CC BY-NC-SA 3.0 IGO), the classification do not use culture; instead of this, leprosy can be classified on the basis of clinical manifestations and skin smear results. In the classification based on skin smears, patients showing negative smears at all sites are said to have paucibacillary leprosy (PB), while those showing positive smears at any site are said to have multibacillary leprosy (MB). Smear microscopy is an easy-to-perform and low-cost procedure, allowing any laboratory to perform it.

Initial skin smears are usually taken from 6 "routine sites" (both earlobes, elbows, and knees) as well as several typical lesions from the patient. Repeat smears are obtained from 3 to 4 of the most active sites previously tested to evaluate progress.

The technique consists of collecting dermal scrapings from the lesions of the patient diagnosed with leprosy. The dermal scraping collected from the lesions must be spread on the surface of a slide in a thin and uniform layer. The blade must remain in a flat place at room temperature until it is completely dry. After the slide is completely dry, it is fixed by passing it quickly through the flame of an alcohol lamp or gas burner three times, and then staining is performed using the cold Ziehl-Neelsen technique and the slide is read under a microscope. It is a complementary analysis to clinical evaluation.

#How mice were handled during preparation of antigen? Were they killed? If yes describe method of killing?

A total of thirty (30) animals were used to produce *Toxoplasma gondii* antigens. However, STAg (Soluble *Toxoplasma gondii* Antigens) are produced for serology analysis used by several projects in our research group. The animals were euthanized by use of an excessive dose of Urethane anesthetic. Our animal protocol for the production of STAg was approved in 2018 and

revalidated in 2023 by the Animal Use Ethics Committee from State University of Northern Rio de Janeiro. So we added in Line #195: The animals were euthanized using an excessive dose of Urethane anesthetic and peritoneal fluid containing *Toxoplasma gondii* tachyzoites was obtained by peritoneal lavage.

And in Line #200: Animal handling was conducted in accordance with the guidelines of the National Council for the Control of Animal Experimentation and international recommendations. Experimental protocols for STAg production were approved by Animal Use Ethics Committee from State University of Northern Rio de Janeiro under protocol number 385.

#During recruitment of study participants, Have you considered patients previous condition such as asthma, allergy, parasitic infection which may increase level of IgE and may affect your findings?

No. IgE levels against antigens from intestinal parasites commonly related to poor living conditions, allergies and asthma did not consider in this study. The dosage of IgE in the immunological assay (ELISA) in our study was specific against the PGL-1 antigen from *M. leprae*. So, it certainly did not affect our findings.

So, we rewrote the following that we thought might be confusing: Line # 380 - The presence of other intestinal parasites, allergies and asthma should be also considered because these diseases could trigger Th-1 or Th-2 immune response components and may indirectly influence in the magnitude of immune response against *M. leprae*. Nonetheless, this does not prejudice our findings since leprosy patients with negative serology for *T. gondii* are also subject to these same poor living conditions but did not have high levels of IgE against *M. leprae* PGL-1 and association with the development of a leprosy reaction.

Reviewer #1:

Introduction

• RR and ENL abbreviations page 4, paragraph 3, line 100 and 101

They are divided into type 1 reaction, or Reverse Reaction (RR), and type 2 reaction, or erythema nodosum leprosy (ENL), each one presenting specificity inherent to the pathophysiology, clinical picture and therapy [6].

• Please define Virchowian clearly page 6, paragraph 1, line 122

Virchowian is the same of Lepromatous. It means the most severe form of leprosy. In order to standard the writing of this clinical form of leprosy, we substitute Virchowian for Lepromatous in line #115: According to data from previous studies, infection by *T. gondii* presents as a risk factor for the development of leprosy, especially in its most severe form **(Lepromatous)** that a Th2 immunological response predominates. Proliferation of the bacillus occurs, with the occurrence of many lesions and disseminated infiltrations in the skin and nerves. [10].

Av. Alberto Lamego, 2000 - Parque Califórnia - Campos dos Goytacazes/ RJ - 28013-602
Tel.: (22) 27397255 - correio eletrônico: alba@uenf.br

• Repetitions of sentence so it would be better if you rewrite it. page 6, paragraph 1

Toxoplasmosis is a disease caused by the protozoan apicomplexa *Toxoplasma gondii*, an obligate intracellular parasite with cosmopolitan distribution that infects warm-blooded animals [11]. Is one of the zoonosis that most affects humans worldwide and in the vast majority of the cases manifests itself as asymptomatic due to the immunocompetence of individuals [12].

• Percentages should be separated by dot instead of comma (Page 6, paragraph 3, line 138) E.g 65,9%

Line #132 - In Campos dos Goytacazes - RJ, studies were carried out with a sample of 367 individuals where 65.9 % of these showed positive serology for *T. gondii* [14].

• Reactional episodes in patients can occur before, during and after completing MDT, but you studied the co-parasitism with the occurrence of reactional episodes in patients before initiating MDT, why you wanted to do the study on before MDT? Do you have a reason? It would be good if you elaborate it. Page 7, paragraph 2, line 147.

The intention was to find a marker in an individual with leprosy that could be related to the development of a leprosy reaction. MDT administration could influence with immunological parameters.

• Do you have a follow-up plan to study the effect of this co-parasitism with the occurrence of reactional episodes in patients during and after completion of MDT?

Yes. We have started to follow-up the individuals recently.

<u>**Methodology section :**</u>

• Would you define the Madrid classification? Since there are different types of leprosy classification, it would be better if you make it clear (Page 7, paragraph 3, line 157)

We added in Line #152: The Madrid classification (1953) adopts polarity criteria, based on the clinical characteristics of the disease combined with the bacteriological criteria of leprosy. Thus, the polar groups are defined as: tuberculoid (T) and lepromatous (L); the transient and initial group of the disease, the indeterminate form (I); and the unstable and intermediate form, the borderline (B) or dimorphic (D) form (Souza, 1997).

• Neural leprosy patients were excluded from the analysis. Do you have specific reasons to exclude them from the study? (Page 7, paragraph 5, line 166)

There is no consensus regarding the classification of the neural form. In 1952, Wade classified it as an independent subgroup of leprosy, but this generated many discussions, as some authors believe that it is part of the subgroups of the disease. This form does not fit into any of the subgroups, so it must be analyzed separately, as it presents a difficult diagnosis, the clinical manifestations differ from the other forms, as it does not present skin lesions and the bacilloscopy is negative.

Please define the abbreviations:

- **Correct the ENL, Page 8, Figure 2, line 173:** ENL: erythema nodosum leprosy
- **anti-STAg, Page 8, Paragraph 2, line 178:** anti-STAg (anti- Soluble *Toxoplasma gondii* Antigen) IgG
- **The protein concentration in the supernatant (STAg) was determined by Lowry method. Would you please explain the method and also attach it as annex (page 9, Line 201).**

We cited the reference (Lowry OH, Rosebrough NJ, Farr AL, Randall RJ. Protein measurement with the Folin phenol reagent. J Biol Chem. 1951;193:265–275) that we follow to measure the protein concentration of STAg.

Please define the abbreviations:

- **PBST …Page 9, and Line 208:** PBST (PBS [Phosphate Buffered Saline] 1×; 0.05% Tween 20). Then, 100 µl of blocking buffer (PBST, 1% BSA [Bovine Serum Albumin]) was added in each well and incubated for 30 min at 4 °C.
- **BSA… Page 9, and Line 209:** PBST (PBS [Phosphate Buffered Saline] 1×; 0.05% Tween 20). Then, 100 µl of blocking buffer (PBST, 1% BSA [Bovine Serum Albumin]) was added in each well and incubated for 30 min at 4 °C.
- **I think Immunosorption written in the Enzyme Linked Immunosorption Assay (ELISA), should be changed to Immunosorbent assay Page 10, line 224**

Yes. We change to the correct form in line #237 Enzyme Linked Immunosorbent Assay (ELISA) of immunoglobulins: IgA, IgE, IgG1, IgG2, IgG3 and IgG4.

Please define the abbreviation:

• **ABTS stands for …Page 11, Paragraph 1, and Line 242:** We inserted in line #227 (28 mmol/L citric acid, 48 mmol/L dehydrated sodium phosphate, 1 mg/ml ABTS [2,2'-AZINO-BIS(3-ETHYLBENZOTHIAZOLINE-6-SULFONIC ACID) DIAMMONIUM SALT] and 0.003% H2O2).

Results section:

• **Percentages should be separated by dot instead of comma:** OK. We changed all of them.

• **Page 11, Paragraph 2, line 262 and 263:** OK, corrected.

• **Check Table 2 summation:** OK corrected

• **Make corrections, highlighted Page 13, Line 282:** OK. Corrected

Discussion:

• **The cytokine TFN, Page 14, last paragraph, line 344:** TNF-α

• **The predictive value of anti-PGL-1 immunoglobulin levels for leprosy immune reactions is unclear. Please would you make the sentence clear? Which immunoglobulin? Are you referring to IgM or IgE levels? (Page 15, 1st paragraph, Line 347):** Line #359 - There is no clear relationship between levels of any anti-PGL-1 immunoglobulins and predicted development of a leprosy reaction.

• **Finally, high levels of TNF-a and NO mediated through IgE generation may also account for deleterious chronic inflammatory diseases that are observed during many parasitic infections as leprosy reactions. (Page 15, 1st paragraph, Line 363). The sentence is not clear it seems that leprosy reaction is a parasitic infection, though leprosy is a bacterial infection; leprosy reaction is not! Will you please rewrite it?:** Line #376 - Finally, high levels of TNF-a and NO mediated through IgE generation may also account for deleterious chronic inflammatory diseases that are observed during many parasitic infections and which may be similar to what happens in leprosy reactions.

• **Conclusion: I think this study is the ONLY study done on the increased risk of leprosy reaction with patients that are infected with *T. gondii*, so do you think it is possible to make conclusions as the presence of co-infection with *T. gondii* as high risk to develop leprosy reactions?** *Yes, so we rewrote the following to better*

explain: Our results suggest that *T. gondii* infections may play a role in the progression to more severe type of leprosy reaction, ENL, due to immunomodulation that influences the increase in IgE levels against the *M. leprae* antigen (PGL-1) in *T. gondii* coinfected patients, but not in those not coinfected.

Reviewer #2:

<u>**Materials and Methods**</u>

• The authors should explain more clearly which groups are being studied and what the abbreviations mean. Line 163-166. The paragraph should be placed above on lines 156:

The Madrid classification (1953) adopts polarity criteria, based on the clinical characteristics of the disease combined with the bacteriological criteria of leprosy. Thus, the polar groups are defined as: tuberculoid (T) and lepromatous (L); the transient and initial group of the disease, the indeterminate form (I); and the unstable and intermediate form, the borderline (B) or dimorphic (D) form (Souza, 1997).

• Figure 2. Says study flow chart. There is an error it should say Figure 1: it was changed for Figure 1.

• Table 1. It does not provide further information. What is mentioned in the table could well go in the text: table 1 was removed and added the following text: Line #176 – From the total number of patients recruited, 180 (77 patients with leprosy reaction and 103 without leprosy reaction) were analyzed for the detection of anti-STAg (anti- Soluble *Toxoplasma gondii* Antigen) IgG antibodies and from these, 94 were analyzed regarding the immunoglobulin levels (IgA, IgE, IgG1, IgG2, IgG3 and IgG4) against anti-PGL-1 from *M. leprae*.

• Line 172. It says that not all participants were included in the laboratory studies. Describe how the selection was made, and what criteria were used:

The difference in the number of samples between the tests was due to the insufficient amount of reagents (antibodies) available to carry out the analysis of Igs anti-PGL-1 in all samples. The choice of samples was random for each clinical group. So we added in Line #180 - The difference in the number of samples between the tests was due to the insufficient amount of reagents (antibodies) available to carry out the analysis of Igs anti-PGL-1 in all samples. The choice of samples was random for each clinical group.

<u>**Results**</u>

Table 2. It is poorly tabulated and confusing. See another form of presentation: The Table 2 layout were changed in order promoting understanding.

Figure 2. The authors describe the results of the IGE experiment (lines 287-291), however there are other results that are not explained, such as, for example. IgG1 (values are high in

both groups), IgG2 and 4 (large confidence intervals) will be that the groups correspond to a low number of patients, and IgG3 (the average value is high in both groups). All these results must be explained.

The values between the groups do not differ according to statistical analyses. In this sense, we add the following text: Line # 304 - There was no statistical significance in relation to the production levels of the immunoglobulins IgA, IgG1, IgG2, IgG3 and IgG4 between leprosy patients who developed leprosy reaction and those who did not develop a leprosy reaction (Figure 2).

Discussion

Line 329-333. The conclusion regarding *M. lepra*e infection in the foot of the BALB/c mouse is not correct because the infection did not occur due to the immune protection of *T. gondii* but because the mouse is never susceptible to infection with *Myocbacterium leprae*. To affirm that, the authors mus look for a susceptible host like the armadillo.

Infection of the footpad of BALB/c nude mice with a viable *M. leprae* strain is a model used to maintain a viable *M. leprae* strain in laboratories. We ourselves make use of this *M. leprae* strain maintenance model in our laboratory. However, the authors of the cited work used an immunocompetent Balb/c mouse chronically infected with *T. gondii* to infect the footpad with a viable strain of *M. leprae* and observe the relationship of susceptibility and resistance related to the amount of *M. leprae* bacilli recovered from footpad. So, as this work referenced by us in the discussion was the only one in the scientific literature that showed a model of *M. leprae*/*T. gondii* co-infection, we use the author's conclusion in our discussion. The phrase *“Contrary to our data, BALB/c mice chronically infected with the intracellular protozoan T. gondii or Besnoitia jellisoni were resistant to footpad challenge with M. leprae. Resistance was manifested by lower numbers of recoverable M. leprae in the footpads of protozoal-infected mice and resistance was enhanced in Toxoplasma-infected mice by a booster injection of Toxoplasma antigen in the infected footpad (Krahenbuhl JL, Levy L, Remington JS. Resistance to Mycobacterium leprae in Mice Infected with Toxoplasma gondii and Besnoitia jellisoni. Infect Immun. 1974 Nov;10(5):1068-71. doi: 10.1128/iai.10.5.1068-*

1071.1974. PMID: 16558091; PMCID: PMC423063.)" used in our discussion was not our conclusion, it was from the authors of the work we cited. We transcribe their conclusion in the context of our discussion even using the same words.

Comments

The authors make conclusions on a single biological marker. They should, as far as possible, associate the symptoms and clinical signs of each of the patients (Madrid Classification) with the respective markers and should monitor the marker (IgG E) over time at the time of detection and during treatment to effectively determine that immunomodulation is exerted.

We included in Line #391: Our results suggest that *T. gondii* infections may play a role in the progression to more severe type of leprosy reaction, ENL, due to immunomodulation that influences the increase in IgE levels against the *M. leprae* antigen (PGL-1) in *T. gondii* coinfected patients, but not in those not coinfected.

We also have added in Line #569 the following:

Supporting information

S1 File. Underlying Data Set. The spreadsheet called "Immunoglobulins" refers to the index values used in analyzing the levels of anti-PGL1 immunoglobulins. The worksheets called "Leprosy No Reaction", "Reverse Reaction". "Erythema Nodosum Leprosy" and "RR-ENL" present values used for risk analysis calculations.

Sincerely,

Alba Lucínia Peixoto Rangel

Alba Lucínia Peixoto-Rangel, PhD – corresponding author
Professor, Recognized Biology Laboratory
State University of Northern Rio de Janeiro
