# Supplementary material for: Toxoplasma gondii infection and high levels of IgE are associated to erythema nodosum leprosy (ENL)
Source: PLoS One. 2024 Jun 12;19(6):e0300704. doi: 10.1371/journal.pone.0300704 (PMC11168690; doi:10.1371/journal.pone.0300704)
Supplement: Attachment — Submitted filename: Response to Reviewers.pdf [file pone.0300704.s003.pdf]

Dr[a]. Alba Lucínia Peixoto Rangel
State University of Northern Rio de Janeiro
Bioscience and Biotechnology Center
Recognized Biology Laboratory
Alberto Lamego Avenue, 2000
Campos dos Goytacazes, RJ, Brazil
28013-602
alba@uenf.br

Drª Musa M. Ali
Academic Editor
Plos One

February 01, 2024

Dear *Plos One* Academic Editor:

I am pleased to submit our revised manuscript entitled "***Toxoplasma gondii* infection and high levels of IgE are associated to erythema nodosum leprosy (ENL)**" for publication as Research Article in the *Plos One*. Also, we would like to thank editor and reviewers for comments. Follow below our Response to Editor and Reviewers:

Editor Comments:

All citation in the text was reviewed for reference list as required.

Reviewer #1:

1) I would like the authors to thank for incorporating the comments. I would appreciate if you include your responses to the manuscript so that readers will also understand the reason why you chose to do the study before MDT and also why you excluded the neural leprosy cases.

We included in Line 170 the following: Patients with leprosy relapse and neural leprosy were excluded from the analysis due to there is no consensus regarding the classification of the neural form. In 1952, Wade classified it as an independent subgroup of leprosy, but this generated many discussions, as some authors believe that it is part of the subgroups of the disease. This form does not fit into any of the subgroups, so it must be analyzed separately, as it presents a difficult diagnosis, the clinical manifestations differ from the other forms, as it does not present skin lesions and the bacilloscopy is negative.

We incuded in Line 165 the following: "…since MDT administration could influence with

immunological parameters."

2) RR and ENL abbreviations page 4, paragraph 3, line 99 and 100.

Line 103 - They are divided into type 1 reaction, or Reversal Reaction (RR), and type 2 reaction, or Erythema Nodosum Leprosum (ENL), each one presenting specificity inherent to the pathophysiology, clinical picture and therapy [6].

3) Please rewrite correctly the name of the chemical compound Page 11, Paragraph 1, line 242 (2,2' Azinobis acid; 3-ethylbenzothiazolin-6-sulfonic acid) diammonium salt (Sigma -Aldrich).

Line 257 - After this period, the wells were washed three times with Tris/Tween 0.05% 15mM (pH 7.5) and ABTS substrate solution (2,2' Azinobis acid; 3-ethylbenzothiazolin-6-sulfonic acid) diammonium salt (Sigma -Aldrich) was added to reveal and kept in the dark for 15 to 20 minutes. The plates were read using a plate reader (EPOCH-BioTek) and the 405nm filter was used.

4) Page 13: Table 2 line 293
Please correct the abbreviations RR and ENL as mentioned above.

Line 300: (OR) Odds ratio; (CI) Confidence Interval; (RR) Reversal Reaction; (ENL) Erythema Nodosum Leprosy; (RR/ENL) two reactions at the same time. Fisher's test for analyzes; *Statistical Significance.

Reviewer #2:

Materials and methods

1) **Line 151. Fig 1 must be placed in parentheses as in Line 160. (Fig 1),Line 301 (Fig 2) Line 304 (Fig 2), Line 306 (Fig 2b), Line 329 (Fig 3)**

   All citation of figures was put in parenthesis.

2) **Line 152-157. The description of the Madrid classification is confusing. It is not only a histological classification but includes four aspects: clinical, baciloscopic, immunological and histopathological. It comprised two types Tuberculoid (T) and Lepromatous (L) and two unstable groups Indeterminate (I) and Domorphic or Borderline (D/B). (Souza, 1997). This cite must be added to the bibliopgraphy.**

Madrid classification, used in this work, does not use immunological and histopathological parameters. It uses only clinical and bacilloscopic parameters. The classification that uses immunological and histopathological parameters, beyond clinical and bacilloscopic, is the Ridley Jopling classification. So, in order to better explain the Madrid Classification, we added in **Line 156** the following: The Madrid classification is based on clinical and bacilloscopic characteristics, dividing leprosy into two unstable groups, indeterminate and dimorphic or borderline, and two stable types, tuberculoid and lepromatous polar.

3) Line 171. Figure 1. Flow chart of study. LR: Leprosy Reaction; PB: paucibacillary leprosy without reaction; MB: multibacillary leprosy without reaction; RR: reversal reaction; ENL: erythema nodosum leprosy; RR/ENL two reactions at the same time; LR Before: patients diagnosed with reactions at the first clinic visit; LR After: patients that developed reaction after follow-up. In this case, must be refered only as PB: paucibacillary clinical form and MB: multibacillary clinical form of the disease. (With reaction and without reaction must be eliminated, because those abbreviations are not in the figure). RR/ENL must be refered as: Reverse Reaction and Eritema Nodosum Leprosy that occurs simultaneously, in order to be consistent with the tables that follow.

Line 176: **Figure 1**. Flow chart of study. LR: Leprosy Reaction; PB: paucibacillary; MB: multibacillary; RR: reversal reaction; ENL: erythema nodosum leprosy; RR/ENL two reactions at the same time; LR Before: patients diagnosed with reactions at the first clinic visit; LR After: patients that developed reaction after follow-up.

4) Line 175. Correspond to the text of the Table 1 deleted. However the text is not clear enough regarding the number of patients analyzed.
From the total number of patients recruited, 180 (77 patients with leprosy reaction and 103 without leprosy reaction) were analyzed for the detection of anti-STAg (anti- Soluble Toxoplasma gondii Antigen) IgG antibodies and from these, 94 were analyzed regarding the immunoglobulin levels (IgA, IgE, IgG1, IgG2, IgG3 and IgG4) against anti-PGL-1 from M. leprae. The difference in the number of samples between the tests was due to the insufficient amount of reagents (antibodies) available to carry out the analysis of Igs anti-PGL-1 in all samples. The choice of samples was random for each clinical group.
This paragraph could be rewritten as: Of a 180 patients; 77 had leprosy reaction and 103 had not. Of this total, how many patients were checked with anti-STAg IgG ? in reference to the text that follows and which says "The difference in the number of samples between the tests was due to the insufficient amount of reagents (antibodies) available to carry out the analysis of Igs anti-PGL-1 in all samples" and that refers the current Table 1? . In this case the Table 1 must be added to the text.

We changed in Line 180 the following: From 180 patients, 77 patients developed leprosy reaction and 103 not developed leprosy reaction. All of these patients were analyzed for the detection of anti-STAg (anti- Soluble *Toxoplasma gondii* Antigen) IgG antibodies. Of 180 patients, 94 were analyzed regarding the immunoglobulin levels (IgA, IgE, IgG1, IgG2, IgG3 and IgG4) against anti-PGL-1 from *M. leprae* (Table 1). The difference in the number of samples between the tests was due to the insufficient amount of reagents (antibodies) available to carry out the analysis of Igs anti-PGL-1 in all samples. The choice of samples was random for each clinical group.
We also included again the Table 1.

Results.

5) Table 1. 1. You must justify the title, 2. tabulate columns 4 and 5, 3. add leprosy to column 1 (No Leprosy Reaction), 4. add the references of (RR) Reversal Reaction, (ENL)
OK. It was done.

6) Erythema Nodosum leprosum, (RR/ENL) Reversal Reaction/Erythema Nodosum Leprosy that occur simultaneously. Although it may be redundant, in each of the tables the abbreviations at the footnotes must be clarified.
OK. We changed all of these sugestion.

7) Table 2. At footnote must be added and the order of the words must be changed as (OR) Odds ratio, (CI) Confidential Interval, (RR) Reversal Reaction, (ENL) Erythema Nodosum leprosum, (RR/ENL) Reversal Reaction/Erythema Nodosum Leprosy that occur simultaneously.

Line 300: (OR) Odds ratio; (CI) Confidence Interval; (RR) Reversal Reaction; (ENL) Erythema Nodosum Leprosy; (RR/ENL) two reactions at the same time. Fisher's test for analyzes; *Statistical Significance.

8) Line 312. Figure 2. Immunoglobulins anti-PGL-1 present at (change at by in the) the serum from (change from by of) leprosy patients coinfected (Tx+) or not (Tx -) with T. gondii, who developed (with reaction) or not (without reaction) leprosy reactions. The IgA levels (A), (B) IgE, (C) IgG1, (D) IgG2, (E) IgG3 and (F) IgG4 were measured by ELISA and are presented as index value. The average of each patient were divided by the average of the

Line 318 - Figure 2. Immunoglobulins anti-PGL-1 present in the serum of leprosy patients coinfected (Tx+) or not (Tx -) with T. gondii, who developed (with reaction) or not (without reaction) leprosy reactions. The IgA levels (A), IgE (B), IgG1 (C), IgG2 (D), IgG3 (E) and IgG4 (F) were measured by ELISA and are presented as index value. The average of each patient were divided by the average of the blank of each ELISA plate in order to get an index value.

9) Line 305. Nonetheless, leprosy patients who developed a leprosy reaction and… The paragraph could rewrite as: Nonetheless, leprosy patients who developed leprosy reaction and…

Line 309 - There was no significative differences in the production of IgA, IgG1, IgG2, IgG3 and IgG4 between those who developed leprosy reaction and those who did not develop a leprosy reaction (Fig 2). Nonetheless, leprosy patients who developed leprosy reaction and were also coinfected with *T. gondii* had elevated levels of IgE (Fig 2b)

Discusion

10) I think that rather than focusing on an experimental model, the authors should discuss other pathologies that co-exist with Leprosy infection such as Leishmanissis, tuberculosis, deep mycoses, etc. highly associated with leprosy given the social and economic conditions in which these populations co-habit.

In fact, initially we also thought about writing in this sense, however these works address the issue of co-infection but do not provide much information about the pathological mechanisms. So, we wrote a little

about other coinfection but, because we found immunoglobulin E as a marker for the development of leprosy reactions in individuals coinfected with *T. gondii*, we prioritized focusing our discussion on experimental models in which this marker were involved.

Sincerely,

Alba Lucínia Peixoto Rangel

Alba Lucínia Peixoto-Rangel, PhD – corresponding author
Professor, Recognized Biology Laboratory
State University of Northern Rio de Janeiro
