# Supplementary material for: Toxoplasma gondii infection and high levels of IgE are associated to erythema nodosum leprosy (ENL)
Source: PLoS One. 2024 Jun 12;19(6):e0300704. doi: 10.1371/journal.pone.0300704 (PMC11168690; doi:10.1371/journal.pone.0300704)
Supplement: Attachment — Submitted filename: Response to Reviewers.pdf [file pone.0300704.s004.pdf]

Dr[a]. Alba Lucínia Peixoto Rangel
State University of Northern Rio de Janeiro
Bioscience and Biotechnology Center
Recognized Biology Laboratory
Alberto Lamego Avenue, 2000
Campos dos Goytacazes, RJ, Brazil
28013-602
alba@uenf.br

Drª Musa M. Ali
Academic Editor
Plos One

March 01, 2024

Dear *Plos One* Academic Editor:

I am pleased to submit our revised manuscript entitled "***Toxoplasma gondii*** **infection and high levels of IgE are associated to erythema nodosum leprosy (ENL)**" for publication as Research Article in the *Plos One*. Also, we would like to thank editor and reviewers for comments. Follow below our Response to Editor and Reviewers:

Editor Comments:

All citation in the text was reviewed for reference list as required and we corrected the reference 14 because the authors name was duplicated as well as 2015 Tese de Doutorado. So, it is as following:

14. Vieira, Flavia Pereira. Contaminação ambiental por oocistos de *Toxoplasma gondii* e toxoplasmose de veiculação hídrica sob a perspectiva da vulnerabilidade de aquíferos. 2015. Tese de Doutorado – Universidade Estadual do Norte Fluminense Darcy Ribeiro, Campos dos Goytacazes.

Reviewer #1:

1) Page 13, Line 293 and line 306

Table 2 and 3

Please change Erythema Nodosum leprosy to Erythema Nodosum Leprosum (ENL)

We changed it as required.

Reviewer #2:

2) line. 495. bibliography citation number 16 does not apply with the Madrid classification. You must change it.

We changed the reference 16 in reference list for:

16. Pavani RAB, Tonolli ER, D'Avila SCGP. Histopathological classification and clinical correlation of 50 leprosy cases from a Teaching Hospital, São José do Rio Preto, São Paulo state, Brazil. Medicina (Ribeirão Preto) 2008; 41 (2): 188-95.

3) Table 2. The footnote must be single space

OK. It was adjusted.

4) Regarding the Discussion. If the authors consider that it is not necessary to discuss the clinical aspects, I have to accept it although I consider that it could contribute an interesting aspect to the work.

We added a paragraph at the beginning of the discussion to contribute a little more about this aspect of co-infections and their clinical impact, as requested in the last review.

Sincerely,

Alba Lucínia Peixoto Rangel

Alba Lucínia Peixoto-Rangel, PhD – corresponding author
Professor, Recognized Biology Laboratory
State University of Northern Rio de Janeiro
